# Use of Indocyanine Green in Fluorescence-Guided Resection of Mesenchymal Hamartoma of the Liver: A Report of Two Cases

**DOI:** 10.7759/cureus.92268

**Published:** 2025-09-14

**Authors:** Lillie Jensen, Tamarah Westmoreland, Lili Miles, Teerin Meckmongkol, Adela Casas-Melley

**Affiliations:** 1 Pathology, University of Central Florida College of Medicine, Orlando, USA; 2 Pediatric Surgery, University of Central Florida College of Medicine, Orlando, USA; 3 Pediatric Surgery, Nemours Children's Hospital, Orlando, USA; 4 Pediatric Pathology, Nemours Children's Hospital, Orlando, USA

**Keywords:** fluorescence-guided surgery, general pediatric surgery, hepatic mesenchymal hamartoma, indocyanine green (icg), intraoperative icg, liver hamartoma, liver mesenchymal hamartoma, pediatric liver tumors, pediatric oncology, spy portable handheld imaging

## Abstract

Indocyanine green (ICG) is a fluorescent agent administered preoperatively to assist in real-time visualization of pathologic tissue during a variety of surgical procedures, including the resection of solid tumors. This series describes the clinical presentation, fluorescence-guided resection, and outcome of two pediatric cases of mesenchymal hamartoma of the liver (MHL), a rare benign hepatic tumor. Use of this agent is widely documented in the literature for the management of more common malignant and benign liver tumors, though reporting for MHL is scarce. It is the purpose of this case series to contribute to the growing understanding of ICG’s utility for this rare tumor, illustrated in two pediatric patients. We present the cases of a 21-month-old male patient and an 18-month-old female patient, both presenting with large hepatic masses causing symptoms secondary to compression of neighboring structures, necessitating surgical removal. Fluorescence-guided surgery using ICG was implemented for the precision resection of both neoplasms. This report provides valuable insight into ICG implementation for MHL in two pediatric patients, outlining how the agent safely and effectively facilitated the resection of these neoplasms.

## Introduction

Indocyanine green (ICG) is a fluorescent agent that can be administered preoperatively to assist in tissue visualization for a variety of surgical indications. ICG preferentially accumulates in certain pathologic tissues, allowing for visualization of disease processes including malignancies, inflammation, and necrosis [1]. This agent has been widely applied in abdominal, gynecologic, and urologic surgery with variable benefit on meta-analysis, dependent on the system of interest [2, 3]. ICG is administered intravenously, the water-soluble compound readily binding to plasma proteins such as albumin. Once bound, ICG is highly selective for uptake by hepatocytes (in an otherwise healthy individual) and is eliminated unchanged by the biliary system. Tissue that is damaged or dysfunctional has a limited or impaired ability to excrete this compound once absorbed, resulting in net accumulation of the dye [3]. Predictable uptake and excretion of ICG in different types of tissues allows the surgeon to time the procedure according to when a structure of interest is made visible by the dye.

A commonly implemented pediatric ICG protocol for primary liver tumors is a single intravenous dose of 0.5 mg/kg given 24 hours up to 14 days prior to surgery [4]. The agent concentrates fairly quickly in the liver, with a delay of at least 24 hours necessary to allow adequate washout (excretion) of dye from healthy hepatic parenchyma so that only diseased hepatic tissue remains visible during surgery [5]. With this rationale in mind, this was the protocol implemented in these two cases, though neither case exceeded an interval of 72 hours. The area in which ICG is concentrated is viewed using specialized tools such as the SPY Portable Handler Imager (SPY-PHI) system (Stryker Corp., Kalamazoo, MI), which was used at this facility. The SPY-PHI camera emits light at near-infrared frequencies, resulting in excitation of the dye and subsequent emission of a fluorescent signal that can be displayed in real-time during operation [2]. Use of this technology in conjunction with ICG is widely implemented in fluorescence-guided hepatic surgeries for malignant tumors such as hepatocellular carcinoma and colorectal liver metastases, as well as for benign tumors such as hepatic hemangiomas and hepatic adenomas [6]. For pediatric or very rare hepatic tumors, there remains a need for more descriptions of use to establish clearer indications, protocols, and utility. 

Mesenchymal hamartoma of the liver (MHL) is a rare, benign neoplasm found primarily in children less than two years of age. It can present as a solitary mass at alarmingly large sizes, with significant potential morbidity due to compression of adjacent structures. MHL appears on ultrasound or MRI as a complex cystic mass with variable solid components, this nonspecific appearance necessitating tissue biopsy for definitive diagnosis [7, 8]. Histopathologic analysis will reveal a mixture of benign epithelial components such as branching or dilated bile ducts, as well as a mesenchymal component of scattered spindle cells arranged in a myxoid or collagenous stroma [8]. Resection is curative with a rare chance of recurrence, though intraoperative visualization can be challenging without the use of fluorescent dye due to MHL's indistinguishable gross appearance from surrounding healthy tissue [9]. Despite its well-established utility in other hepatic tumors, the use of ICG for the resection of MHL is scarcely reported due to its rarity as well as its tendency to arise in young children. This case series contributes two important descriptions of ICG use in MHL resection.

## Case presentation

Cases were identified at Nemours Children’s Health in Orlando, FL, with Case 1 presenting in November 2024 and Case 2 presenting in March 2025. Inclusion of both cases was consecutive based on intraoperative use of indocyanine green and the post-procedure diagnoses of mesenchymal hamartoma of the liver.

Case 1

**Figure 1:**
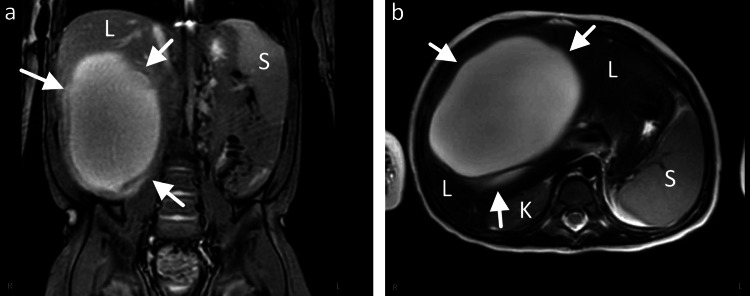
MRI abdomen coronal (a) and axial (b) views of 13.7 x 13.6 x 11.5 cm mixed solid and predominately cystic lesion within the right inferior hepatic lobe L: normal liver; S: spleen; K: kidney; arrows: boundaries of hepatic neoplasm

The patient is a 21-month-old male child with no significant past medical history who presented with a large liver mass identified at an outside hospital. His caretakers reported increased abdominal girth, diminished appetite, and signs of fat loss for one month. The physical examination was notable for a protuberant abdomen with a palpable right-sided mass. The child was in no apparent distress and cooperative throughout the visit. Liver enzymes were markedly elevated, with an aspartate aminotransferase (AST) of >750 U/L and alanine aminotransferase (ALT) of >750 U/L. Alkaline phosphatase and total bilirubin were within normal limits. An abdominal ultrasound was obtained at the outside facility, demonstrating a 13.7 x 13.6 x 11.5 cm mixed, predominantly multi-cystic lesion in the right inferior hepatic lobe. The patient was referred to our center for specialized care. On presentation, an MRI abdomen was obtained to further characterize the lesion. MRI revealed a mixed solid and predominantly multicystic mass, consistent with hepatic mesenchymal hamartoma versus sarcoma (Figure 1a-1b). General surgery was consulted for biopsy versus surgical excision.

Procedure

The decision was made to perform an open, partial right hepatectomy. Resection of the tumor was necessitated by its large size and likelihood of continued expansion, with increased risk of worsening feeding difficulties and respiratory distress. Partial (non-anatomic) lobectomy was chosen in the interest of preserving as much healthy hepatic tissue as possible, as the tumor did not involve the entirety of the right lobe. ICG was administered as a one-time dose of 0.5 mg/kg intravenously 72 hours prior to the procedure.

**Figure 2:**
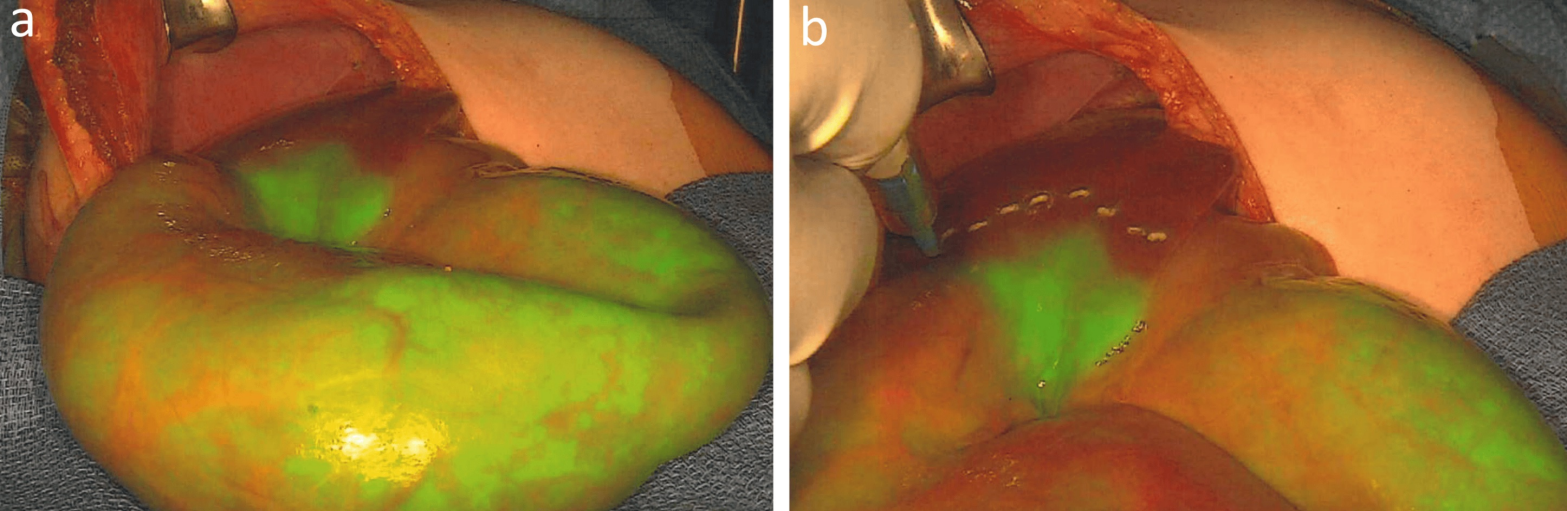
(a) Intraoperative appearance of liver neoplasm displaying strong green fluorescence due to its preferential ICG uptake as seen on overhead monitors using SPY-PHI camera. The hepatic tumor was brought outside the abdominal cavity for visualization. (b) Electrocautery scoring of tumor edges using ICG fluorescence as a guide in order to directly visualize the tumor edge during removal L: adjacent normal liver tissue; dotted line: boundary between healthy and neoplastic tissues; arrows: anatomic orientation, patient lying supine; ICG: indocyanine green

A right subcostal incision was made. Intraoperatively, the SPY-PHI instrument was used to visualize on a screen the extent of ICG dye accumulation, which correlated with the pathologic tissue. The uptake of the dye was clearly identified by a bright green color (Figure 2a), encompassing the gallbladder, liver segments four, five, and six, as well as part of liver segments seven and eight. Using ICG fluorescence as a guide, the liver capsule was scored with electrocautery to identify margin edges with an approximately 2 cm margin outside of the mass visualized on the overhead screens (Figure 2b). Once scored, electrocautery was used to divide the hepatic capsule and subsequently the right hepatic lobe at predetermined sites. Vessels throughout were ligated and sharply divided, and the resected tumor was collected as a fresh specimen for pathology. Residual dye was visualized within the biliary duct, where it was expected to accumulate as it drained from the liver. A Blake drain was placed in the resection bed, and closure occurred without incident. The patient maintained excellent hemostasis throughout the procedure and was transported to the pediatric intensive care unit in stable condition. 

Postoperative Course

**Figure 3:**
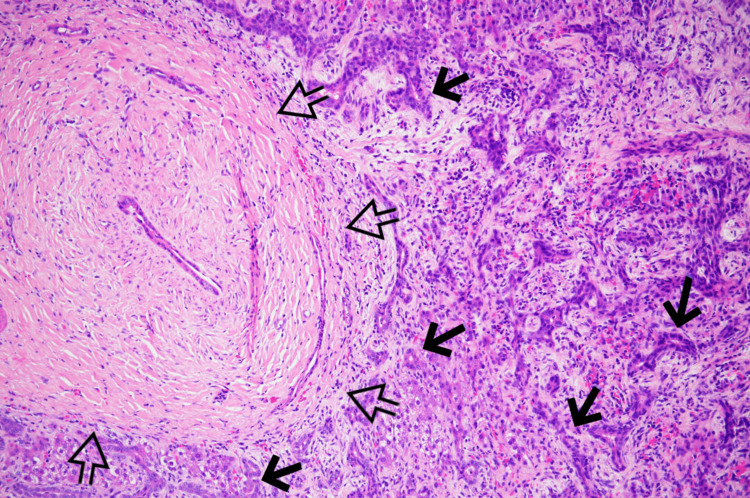
H&E-stained section revealing clusters of branching bile ducts (solid arrows) and fibrocollagenous tissue (empty arrows) in 10X magnification

The postoperative course was free of major or immediate complications. Repeat liver enzymes indicated a relative reduction of AST (60 U/L), ALT (114 U/L), and an anticipated increase of alkaline phosphate (617 U/L). Total bilirubin remained within normal limits. Output from the Blake drain resolved by postoperative day 5, upon which it was removed, and the patient met criteria for discharge. On follow-up in the clinic one week later, the patient’s caretaker reported improved appetite, energy, eating, and stooling. There was no erythema or drainage observed surrounding the well-healing incisions. The pathology report of the solid tumor specimen established a definitive diagnosis of mesenchymal hamartoma of the liver (Figure 3). The patient remained recurrence-free at follow-up eight months later.

Case 2

**Figure 4:**
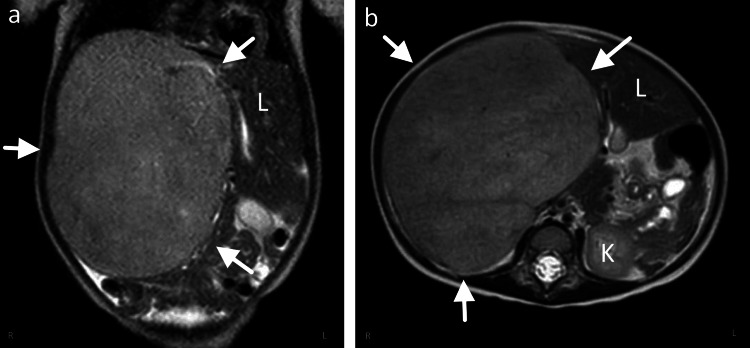
MRI abdomen coronal (a) and axial (b) views of 14.1 x 11.5 x 14.5 cm solid mass involving the anterior and posterior segments of the right hepatic lobe L: normal liver; K: kidney; arrows: boundaries of hepatic neoplasm

The patient is an 18-month-old female child with no significant past medical history who presented as a transfer from an outside hospital with a newly identified liver mass. Her parents noticed abdominal distension and poor appetite for one week, with intermittent subjective fevers and runny stools. They took the patient to her primary care provider, who noted a right-sided abdominal mass and recommended an escalation of care. Physical examination at the subsequent outside hospital visit was notable for a firm, distended abdomen with diffuse tenderness to palpation. The infant was irritable but able to be consoled throughout the visit. Liver enzymes showed mild elevation of AST (59 U/L) while ALT, alkaline phosphatase, and total bilirubin were within normal limits. Abdominal radiographs taken at this facility were significant for gaseous distension with a space-occupying lesion within the abdomen causing mass effect on the bowel. The decision was made to transfer to Nemours Children’s Hospital in Orlando for specialized care, where further imaging and needle biopsy could take place. Abdominal MRI revealed a large, 14.1 x 11.5 x 14.5 cm solid hepatic tumor involving the anterior and posterior segments of the right liver lobe, concerning for neoplastic etiology (Figure 4). Pathological analysis of the submitted core needle biopsy was suggestive of mesenchymal hamartoma of the liver (features described in the postoperative course). General surgery was consulted, and the patient was presented in the facility’s multidisciplinary tumor board for further discussion.

Procedure

The decision was made to perform an open, total (anatomic) right hepatic lobectomy. As in the first case of this series, resection of the tumor was necessary due to the risk of continued expansion and compression of adjacent structures. In this case, the decision was also informed by a pre-procedure diagnosis of MHL for which resection is known to be curative. A total (anatomic) lobectomy was chosen due to the tumor encompassing the entire right hepatic lobe. ICG was administered as a one-time dose of 0.5 mg/kg intravenously 36 hours prior to the procedure.

**Figure 5:**
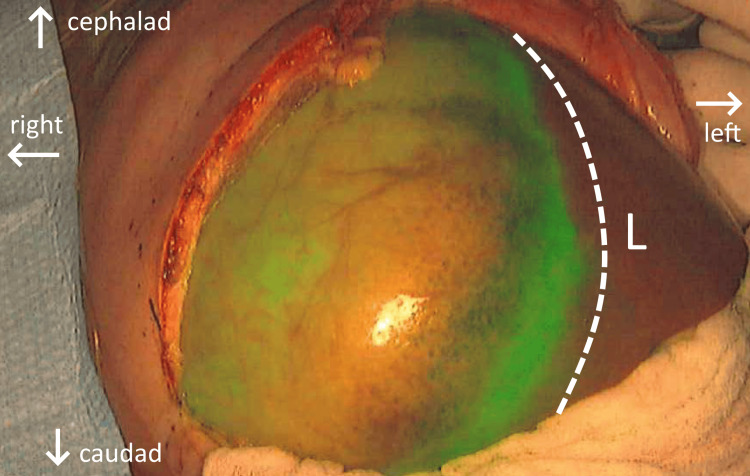
Hepatic neoplasm visible from open surgical incision displaying strong green fluorescence due to preferential ICG uptake, as seen on overhead monitors using SPY-PHI camera L: adjacent normal liver tissue within abdominal cavity; dotted line: boundary between healthy and pathologic liver tissue; arrows: anatomic orientation, patient lying supine

A Chevron incision was made and carried down into the abdomen. The SPY-PHI instrument was used intraoperatively to highlight the extent of the tumor via green fluorescence (Figure 5). The sharply demarcated tumor edges seen on overhead monitors were traced using a surgical ink pen. The limits of the tumor were visualized right at the boundary between the right and left lobes. The tumor was precisely excised following this boundary and collected as a fresh specimen for pathologic analysis. Following right lobectomy, the SPY-PHI camera indicated further involvement of a portion of the caudate lobe, which was also removed. A Blake drain was placed adjacent to the transected liver surface and around the porta hepatis. To prevent torsion and vascular compromise of the liver remnant, the falciform ligament was reattached. Closure occurred without incident, and the patient was transported to the PICU in stable condition.

Postoperative Course

**Figure 6:**
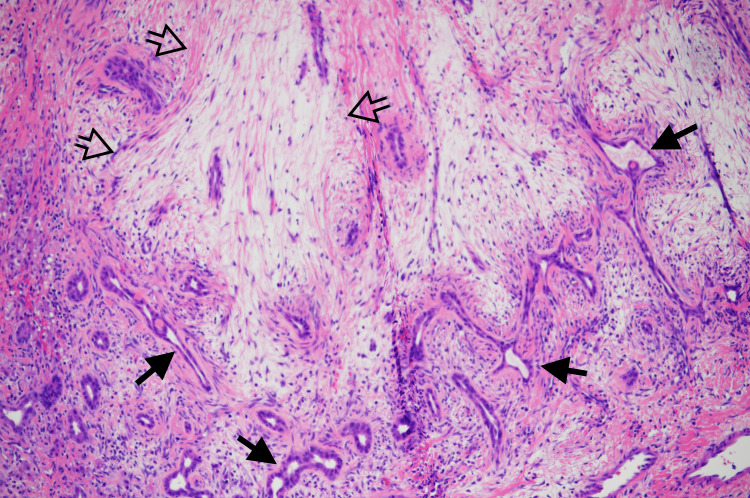
H&E-stained section revealing predominantly solid liver tumor with many irregularly shaped bile ducts (solid arrows) admixed with myxoid stroma (empty arrows) in 10X magnification

The postoperative course was free of major or immediate complications. Extubation was achieved on postoperative day 3, and recovery proceeded uneventfully. Output from the Blake drain was resolved by postoperative day 10 and was removed. Repeat labs showed expected mild elevations in AST (58 U/L) and ALT (43 U/L), and alkaline phosphatase and total bilirubin remained within acceptable ranges. The patient met the criteria for discharge at this time and was sent home with her parents. A follow-up appointment two weeks later was uneventful with no pain, fever, swelling, redness, or drainage from the well-healing incisions. The pathology report of the solid tumor specimen reaffirmed the preoperative (core biopsy) diagnosis of mesenchymal hamartoma of the liver (Figure 6). The patient remained recurrence-free at follow-up three months later. 

A summary of the key clinical features of the cases is provided in Table 1.

**Table 1:** Summary *Administered as a one-time intravenous push; **As of most recent follow-up visit

Patient	Tumor size	Procedure	ICG dose*	Pre-procedure interval	Recurrence-free interval**
21-mo. male	13.7 x 13.6 x 11.5 cm	Partial right hepatic lobectomy	IV, 0.5 mg/kg	72 hours	8 months
18-mo. female	14.1 x 11.5 x 14.5 cm	Total right hepatic lobectomy	IV, 0.5 mg/kg	36 hours	3 months

## Discussion

ICG has a well-established role in pediatric solid tumors, and its ability to image tumors in real-time intraoperatively is advantageous to surgeons. The earliest established use was in hepatobiliary surgical cases, such as for hepatoblastoma, with more recent application in the identification of distant metastases [3, 6]. It is low-cost and widely available, with robust evidence validating an excellent safety profile in the pediatric population with little to no reported complications in systematic reviews [2, 10]. A comprehensive literature review examining ICG’s use in pediatric hepatic tumors found that surgeries utilizing this agent reliably achieved complete resection of neoplastic tissue in 90-100% of operations, though this particular study, spanning 108 cases, does not include any cases of MHL [5]. One Polish multi-center cohort study (2025) spanning 136 fluorescence-guided surgeries over four years details just one case of ICG use for MHL, a reflection of this neoplasm’s rarity and the need for more published accounts such as this series [10].

The limitations of ICG are byproducts of its mechanism, including instances of false positivity up to 35.3% in the identification of hepatic malignancies, most often due to patient characteristics that impair dye clearance in the "normal," or non-neoplastic tissues [5]. Inability for dye to clear the non-tumor hepatic parenchyma creates "background noise," making it difficult to easily distinguish ICG concentration in the tissue of interest. As such, patients with conditions such as cirrhosis may require longer than 24 hours from ICG administration to achieve a similar effect. Other drawbacks of ICG use include poor visualization of deeper (> 10 mm from the surface) tissues, due to the inability of infrared light from fluorescent devices to penetrate tissues beyond this level to excite the dye [4]. In regard to protocol of administration, highly variable dosing and timing are reported throughout the literature, with optimal strategies exceptionally difficult to establish due to significant differences in dye behavior between tissue and tumor types. Adding variables such as the fluorescent imaging system used and individual patient characteristics presents a significant challenge in gathering sufficient data to standardize use. Future attempts to establish specific administration standards would necessitate an extensive collaborative movement to define best practices [6, 10].

The cases presented above implemented an ICG protocol of 0.5 mg/kg pushed intravenously < 14 days prior to surgery, with at least 24 hours elapsing between the one-time dose and operation to allow for adequate dye washout. In both procedures, ICG provided excellent visualization of these large, space-occupying tumors, and in Case 1, allowed for greater preservation of healthy right liver lobe tissue due to the sharp demarcation seen between healthy liver and tumor. In Case 2, the dye indicated total involvement of the right hepatic lobe, necessitating total lobectomy, and would additionally identify a small focus of involvement in the caudate lobe that was not apparent on imaging prior to the procedure. These cases showcase the distinct advantages of real-time imaging with fluorescent dyes, permitting the surgeon to readily adapt to new findings discovered intraoperatively. Both patients had good outcomes, free of adverse effects from ICG, and at the time of this article, had no recurrence of their tumors on follow-up visits. These accounts serve to inform pediatric surgeons who are either still considering bringing ICG into their practice or who intend to fine-tune their own protocols for those rare solid tumors where ICG use is less documented.

The limitations of this series are those inherent to case reports, such as a small sample size (N = 2). As a single-center study, it is exceedingly difficult to observe a sufficient case number for satisfactory observational data, and as such, a multi-center study would be the logical next step in gathering data on ICG use in rare pediatric solid tumors. Another limitation is selection bias, as those studies chosen for case reports often represent extreme presentations of a disease (in this case, the large size of the tumor), and as such outcome cannot be generalizable to all cases.

## Conclusions

This case series outlines the use of ICG for fluorescence-guided resection of mesenchymal hamartoma of the liver in two pediatric patients, providing valuable insight into pediatric protocol. This series outlines in specific detail the dosing, timing, and operative strategy in which ICG safely and effectively facilitated tumor resection, informing clinicians of the agent’s utility and practical implementation in this rare hepatic neoplasm.
